# Supplementary figures and images for: Modern-day SIV viral diversity generated by extensive recombination and cross-species transmission
Source: PLoS Pathog. 2017 Jul 3;13(7):e1006466. doi: 10.1371/journal.ppat.1006466 (PMC5510905; doi:10.1371/journal.ppat.1006466)

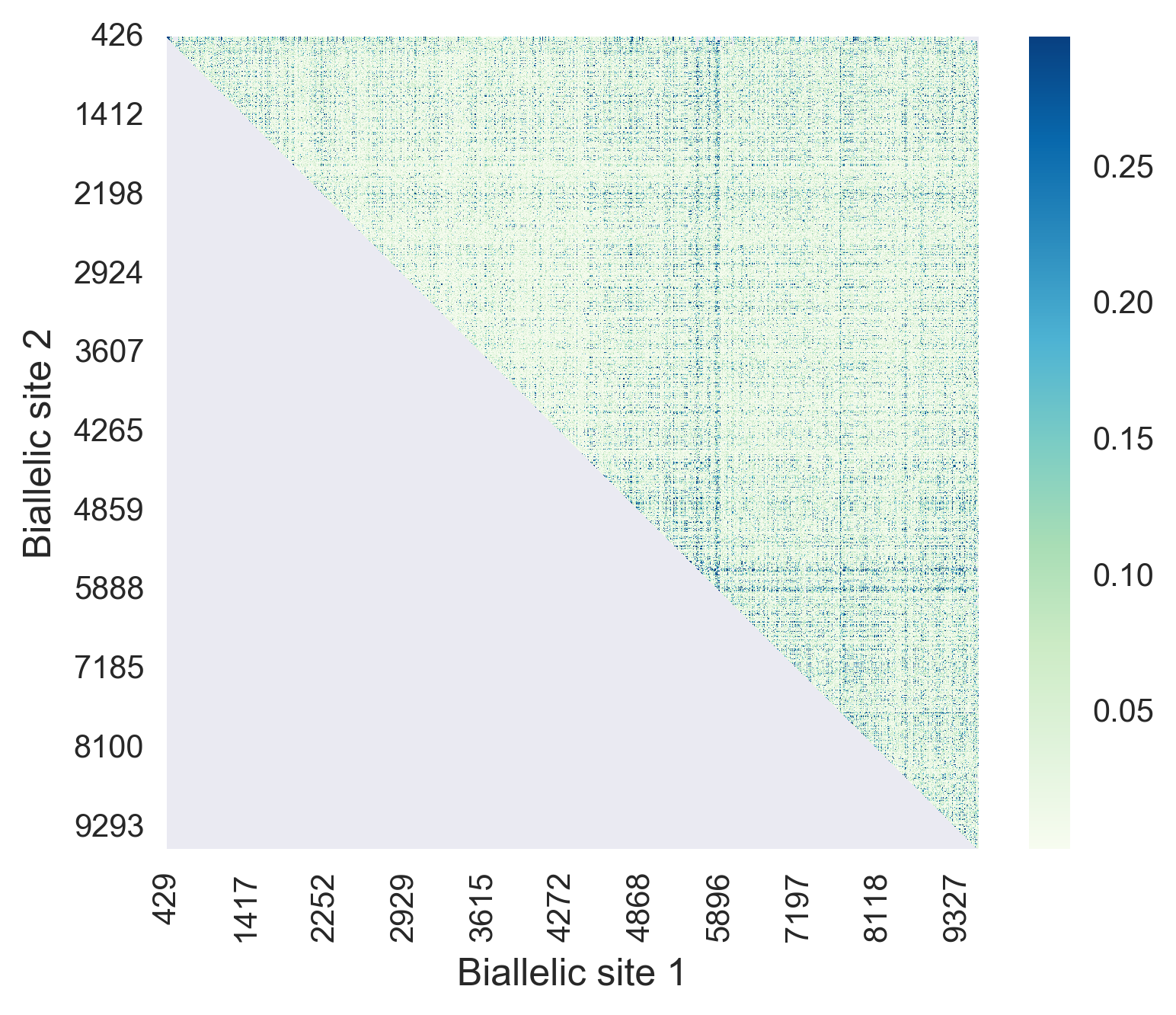

Supplement: S1 Fig — For pairs of biallelic sites (ignoring rare variants), R^2 was used to estimate how strongly the allele in one site predicts the allele in the second site, with values of 0 indicating no linkage and 1 indicating perfect linkage. The mean value of R^2 was 0.044, indicating very low levels of linkage overall. (TIF) [file ppat.1006466.s001.tif]

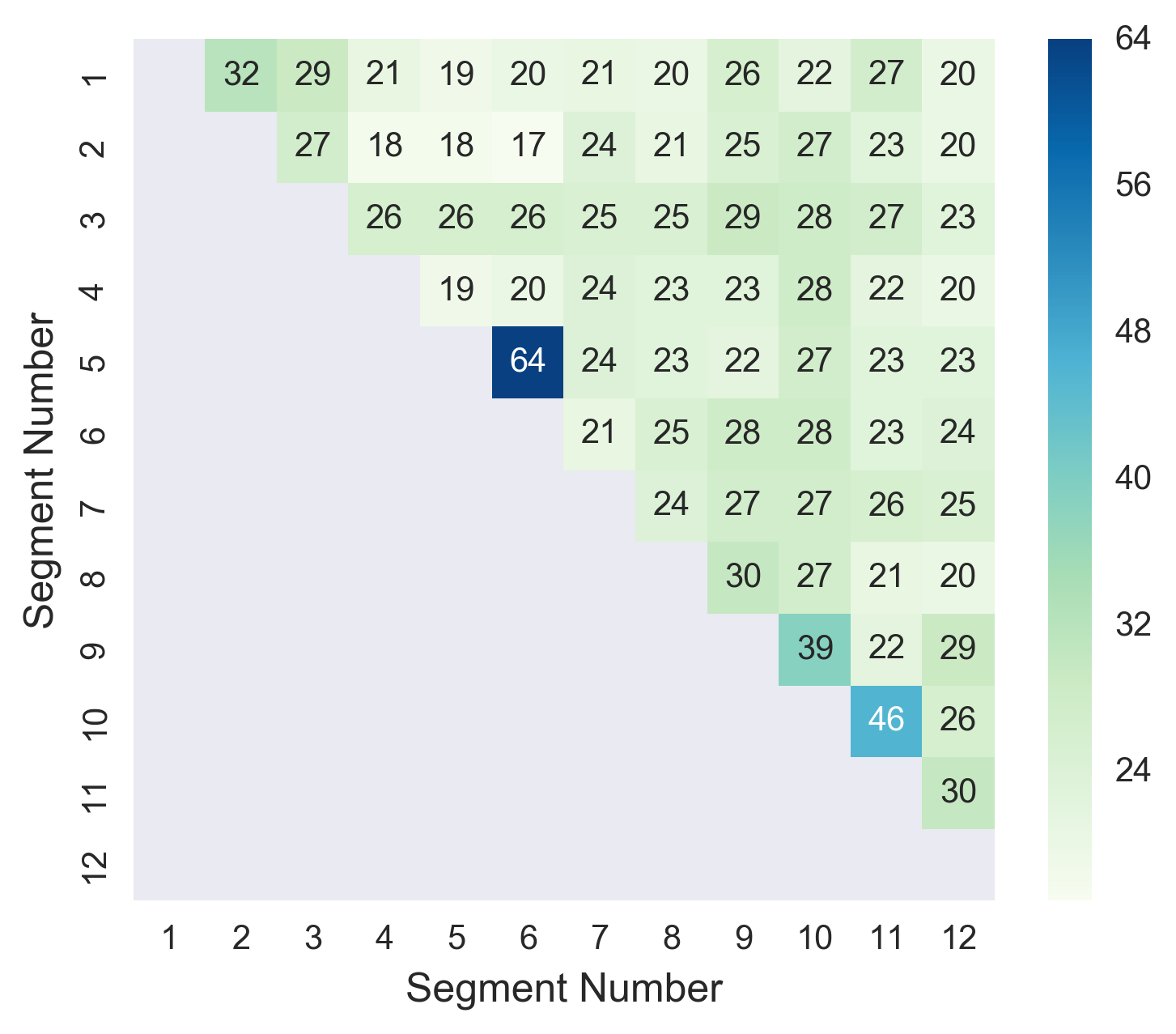

Supplement: S2 Fig — The alignment used for GARD analyses (LANL compendium with HIV overrepresentation reduced) was split along the breakpoints identified by GARD to yield the 12 genomic segments, and a maximum likelihood tree was constructed for each. The number of steps required to turn one tree topology into another was assessed for each pair of trees with the Rooted Subtree-Prune-and-Regraft (rSPR) package. Segment pairs with similar topologies have lower scores than segments with less similar topologies. (TIF) [file ppat.1006466.s002.tif]

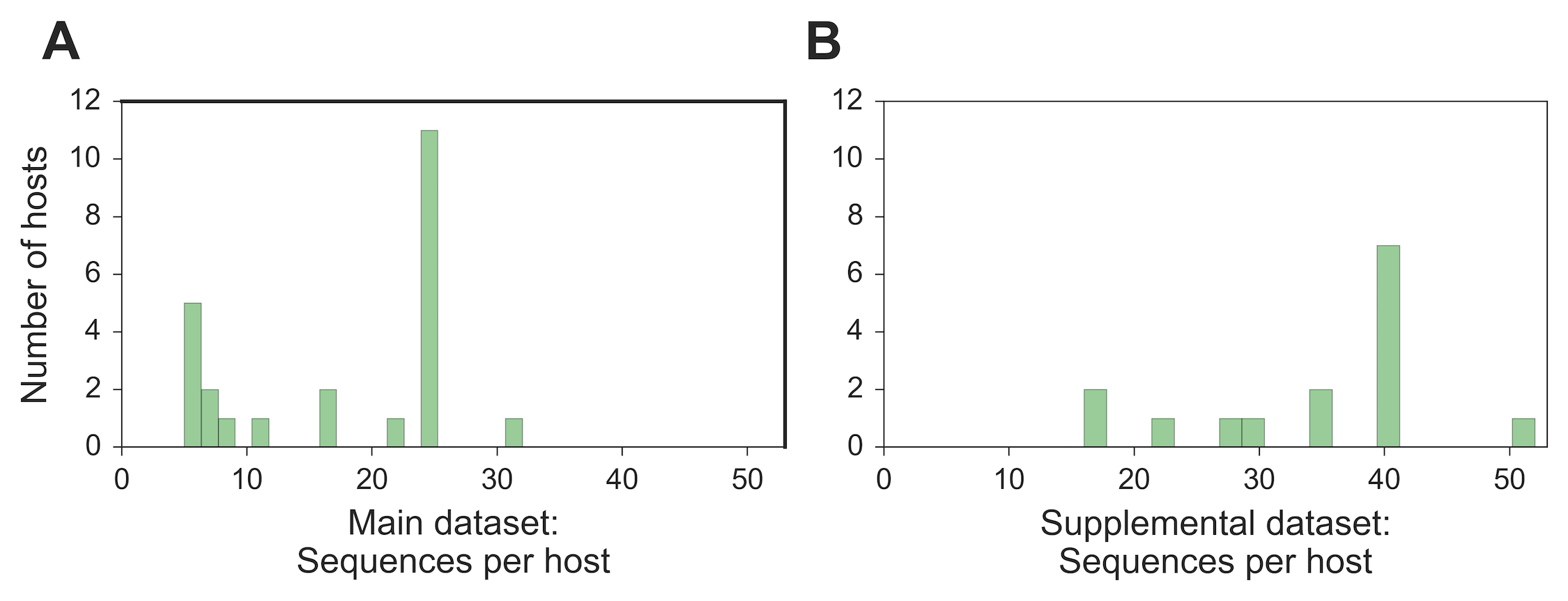

Supplement: S3 Fig — A: All available high-quality lentivirus sequences were randomly subsampled up to 25 sequences per host for the main dataset. We included the 24 hosts with at least 5 sequences available in this dataset. B: For the supplemental dataset, we randomly subsampled up to 40 sequences per host, and included the 15 hosts with at least 16 sequences available in this dataset. For both datasets, a small number of additional sequences were permitted for the few hosts that are infected by multiple viral lineages in order to represent the full breadth of known genetic diversity of lentiviruses in each host population. (TIF) [file ppat.1006466.s003.tif]

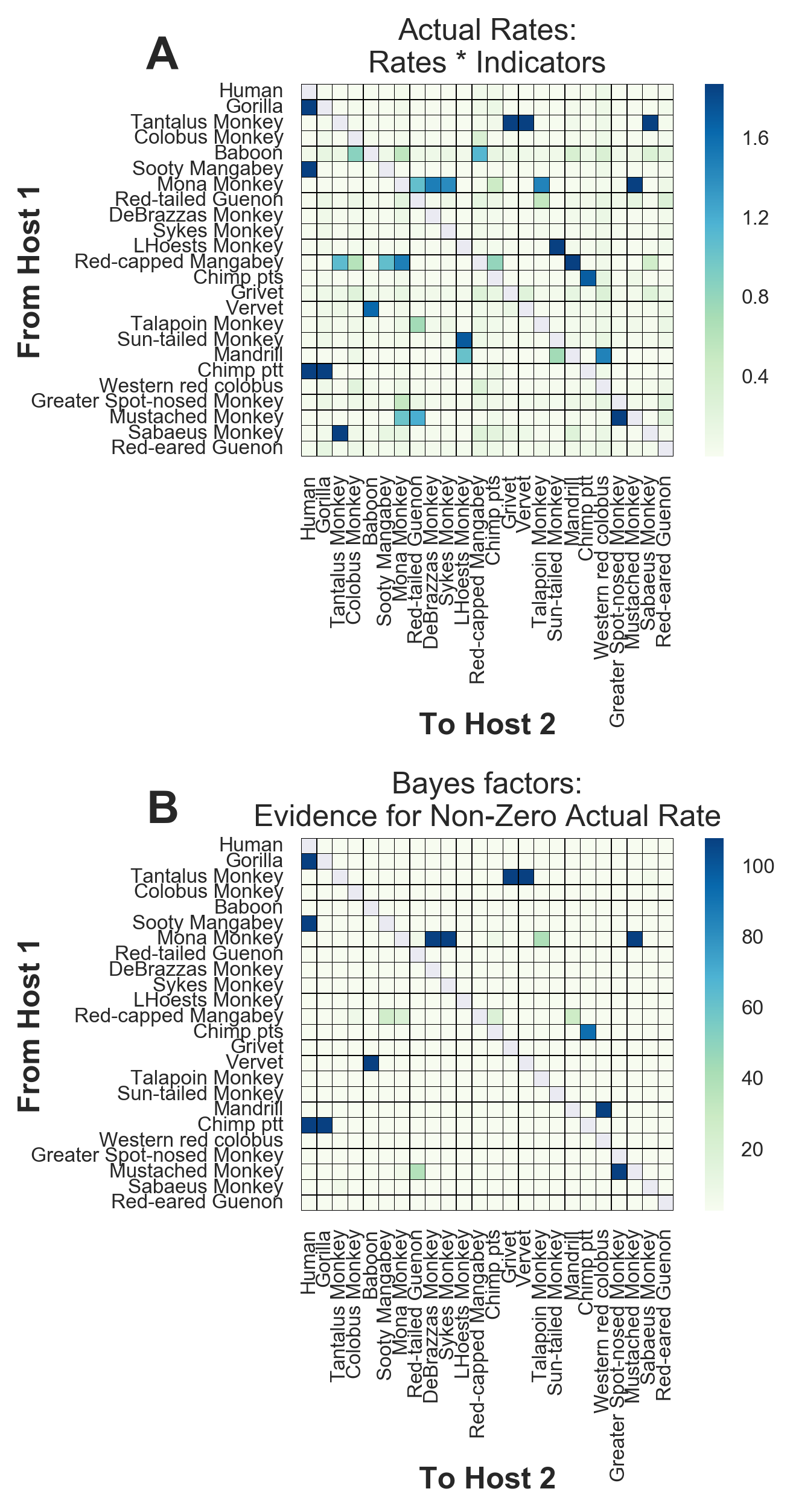

Supplement: S4 Fig — Values for the asymmetric transition rates between hosts, as estimated by the CTMC, were calculated as rate × indicator (element-wise for each state logged). We report the average posterior values above. Bayes factors represent a ratio of the posterior odds / prior odds that a given actual rate is non-zero. Because each of the 12 segments contributes to the likelihood, but they have not evolved independently, we divide all Bayes factors by 12 and report the adjusted values above (and throughout the text). (TIF) [file ppat.1006466.s004.tif]

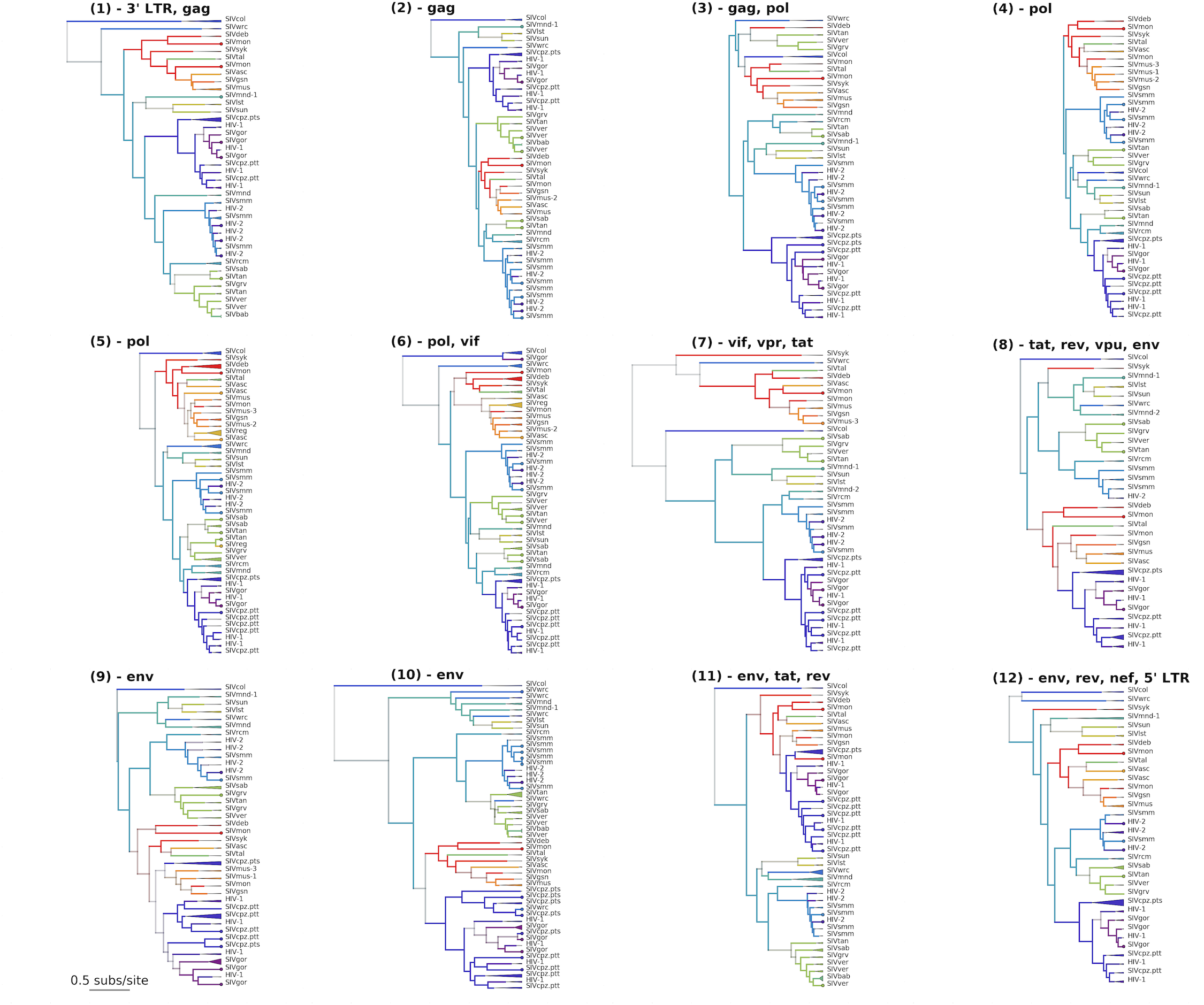

Supplement: S5 Fig — Tips are color coded by known host state; branches and internal nodes are color coded by inferred host state, with color saturation indicating the confidence of these assignments. Monophyletic clades of viruses from the same lineage are collapsed, with the triangle width proportional to the number of represented sequences. (TIF) [file ppat.1006466.s005.tif]

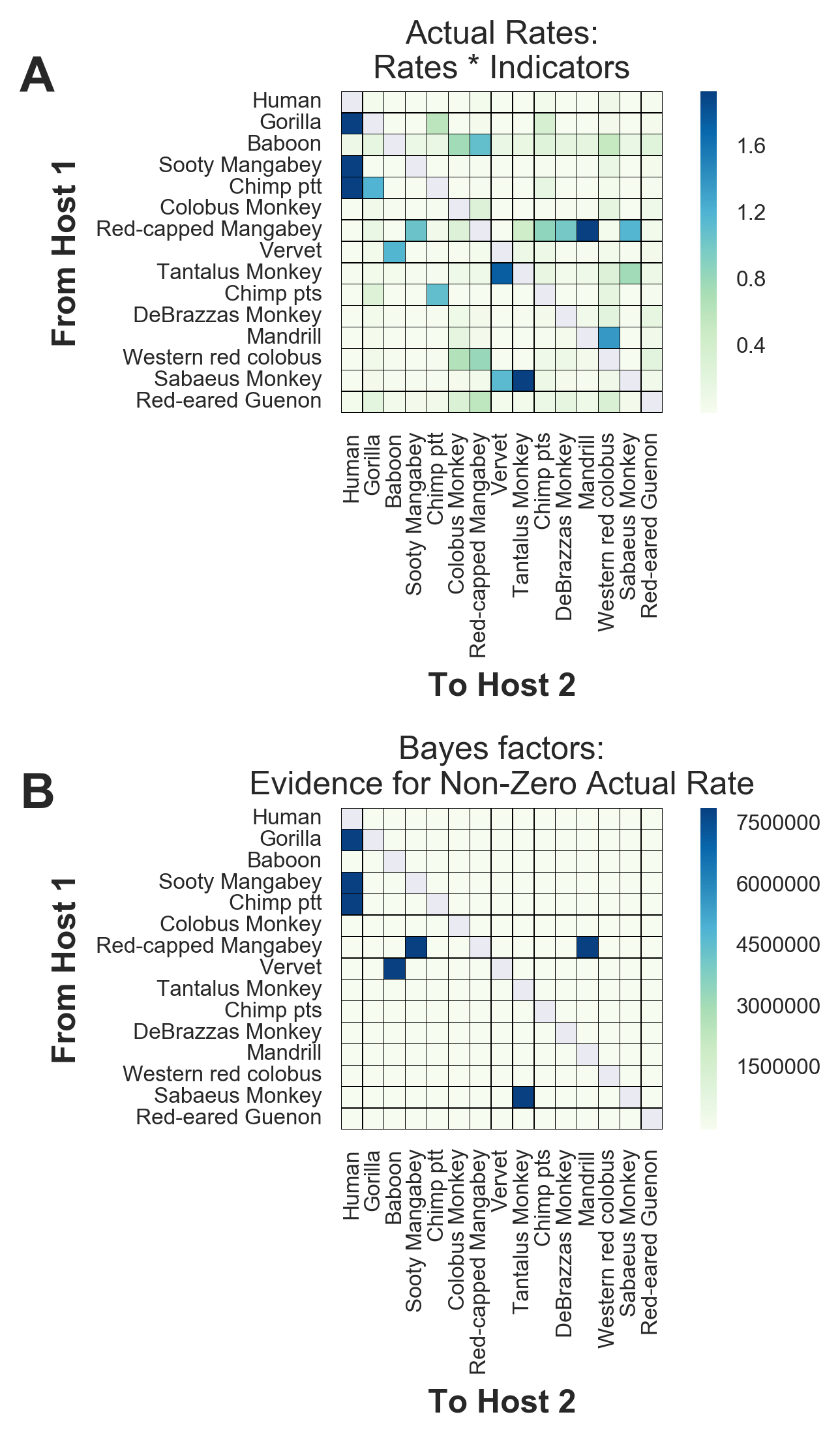

Supplement: S6 Fig — Values for the asymmetric transition rates between hosts, as estimated by the CTMC, were calculated as rate × indicator (element-wise for each state logged). We report the average posterior values above. Bayes factors represent a ratio of the posterior odds / prior odds that a given actual rate is non-zero. Because each of the 12 segments contributes to the likelihood, but they have not evolved independently, we divide all Bayes factors by 12 and report the adjusted values above (and throughout the text). (TIF) [file ppat.1006466.s006.tif]

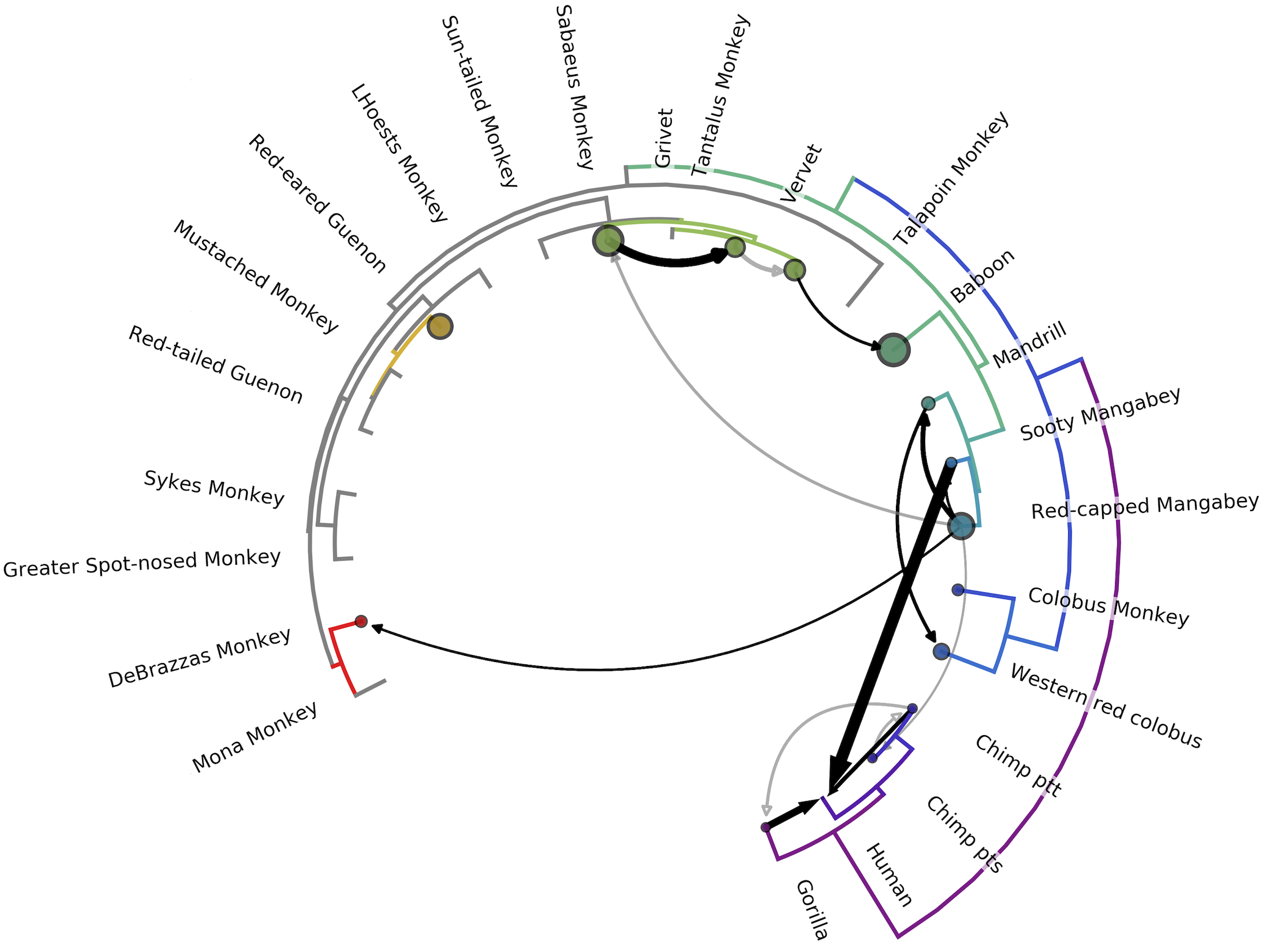

Supplement: S7 Fig — The phylogeny of the host species' mitochondrial genomes forms the outer circle. Arrows with filled arrowheads represent transmission events inferred by the model with Bayes' factor (BF) ≥ 3.0; black arrows have BF ≥ 10, with opacity of gray arrows scaled for BF between 3.0 and 10.0. Transmissions with 2.0 ≤ BF < 3.0 have open arrowheads (see Discussion). Width of the arrow indicates the rate of transmission (actual rates = rates × indicators). Circle sizes represent network centrality scores for each host. Transmissions from chimps to humans; chimps to gorillas; gorillas to humans; sooty mangabeys to humans; sabaeus to tantalus; and vervets to baboons have been previously documented. To our knowledge, all other transmissions illustrated are novel identifications. (TIF) [file ppat.1006466.s007.tif]

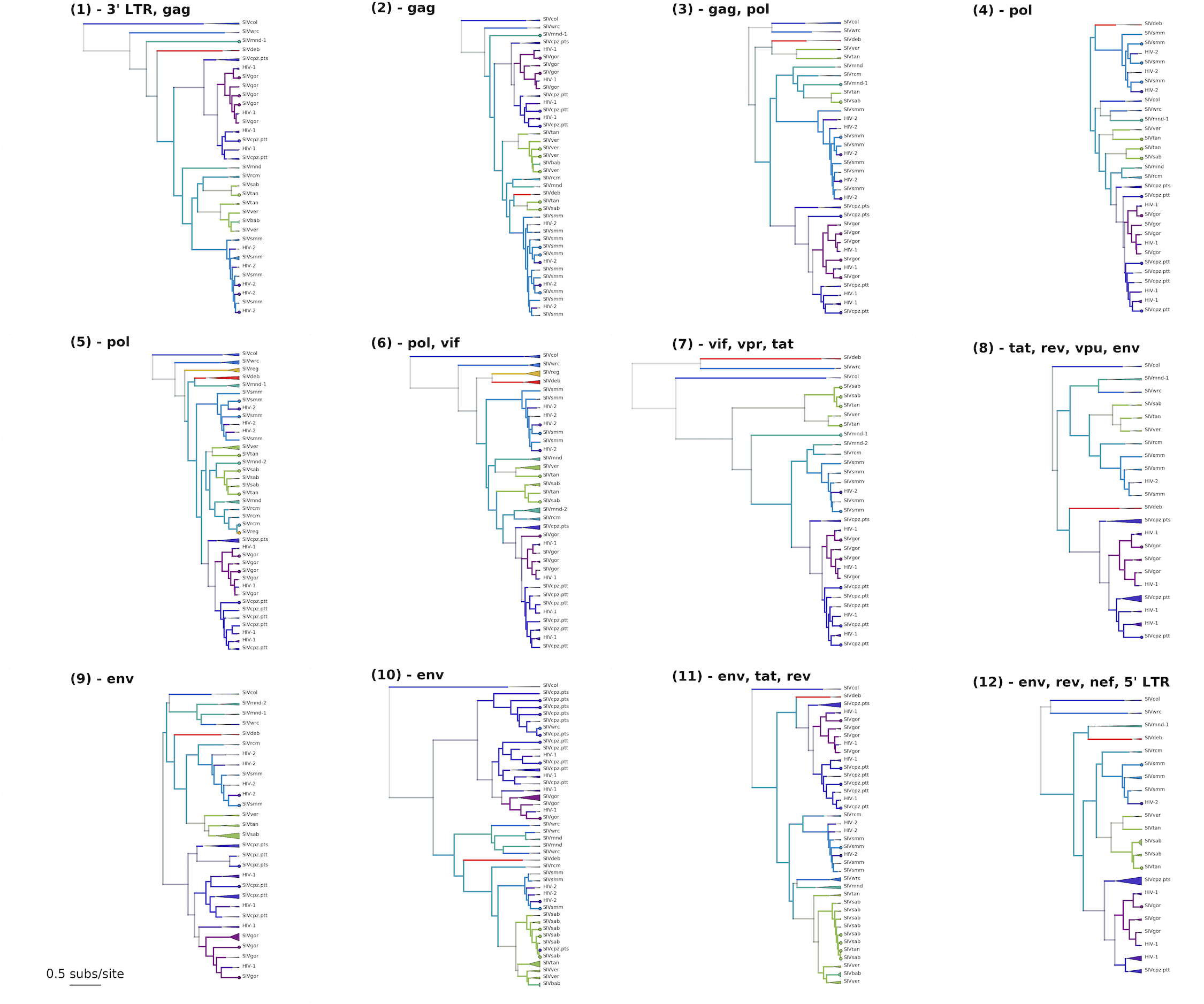

Supplement: S8 Fig — Tips are color coded by known host state; branches and internal nodes are color coded by inferred host state, with color saturation indicating the confidence of these assignments. Monophyletic clades of viruses from the same lineage are collapsed, with the triangle width proportional to the number of represented sequences. (TIF) [file ppat.1006466.s008.tif]

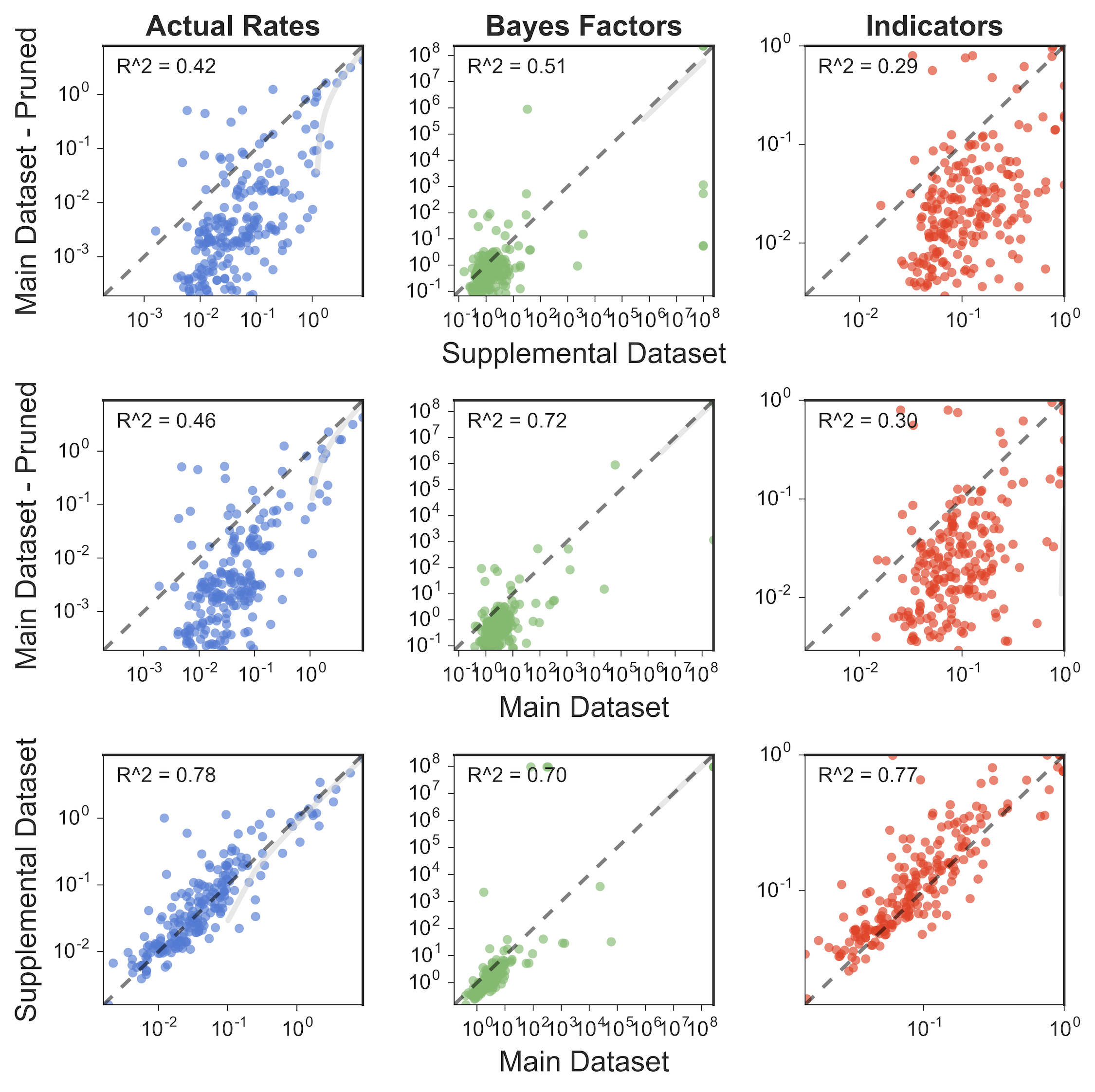

Supplement: S9 Fig — Each datapoint represents on of the 210 possible transmissions between each pair of the 15 hosts present in the supplemental, main, and main/pruned datasets. The main dataset consists of 5–25 sequences from each of 24 host species; the pruned dataset is identical, but with short taxa removed from each segment. The supplemental dataset consists of 16–40 sequences from each of 15 host species. The black dashed line shows y = x; the linear regression is shown in gray. (TIF) [file ppat.1006466.s009.tif]

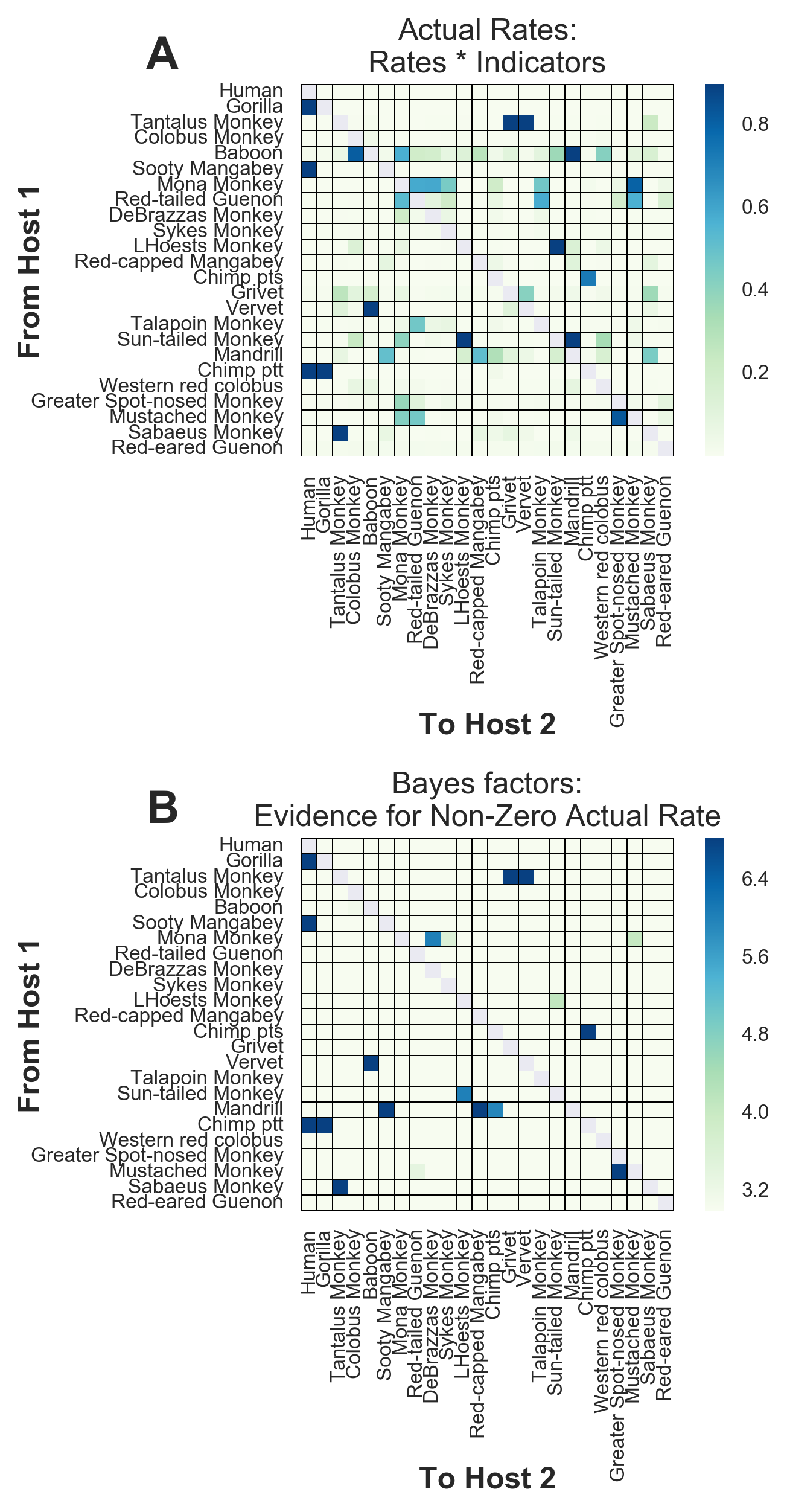

Supplement: S10 Fig — Values for the asymmetric transition rates between hosts, as estimated by the CTMC, were calculated as rate × indicator (element-wise for each state logged). We report the average posterior values above. Bayes factors represent a ratio of the posterior odds / prior odds that a given actual rate is non-zero. Because each of the 12 segments contributes to the likelihood, but they have not evolved independently, we divide all Bayes factors by 12 and report the adjusted values above (and throughout the text). (TIF) [file ppat.1006466.s010.tif]

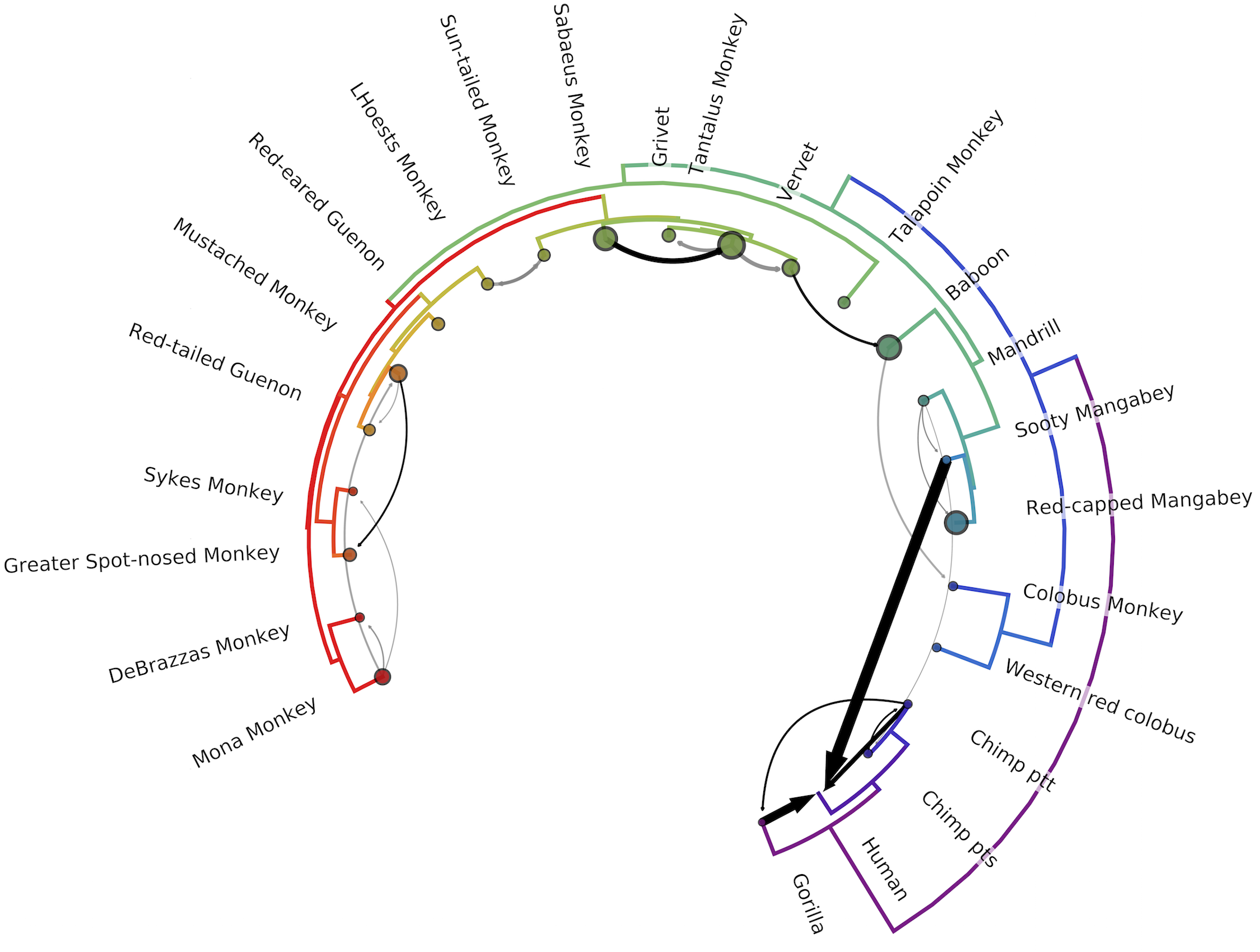

Supplement: S11 Fig — The phylogeny of the host species' mitochondrial genomes forms the outer circle. Arrows represent transmission events inferred by the model with Bayes' factor (BF) ≥ 3.0; black arrows have BF ≥ 10, with opacity of gray arrows scaled for BF between 3.0 and 10.0. Width of the arrow indicates the rate of transmission (actual rates = rates × indicators). As illustrated and discussed, pruning short taxa from the topology can introduce unusual model behavior. (TIF) [file ppat.1006466.s011.tif]

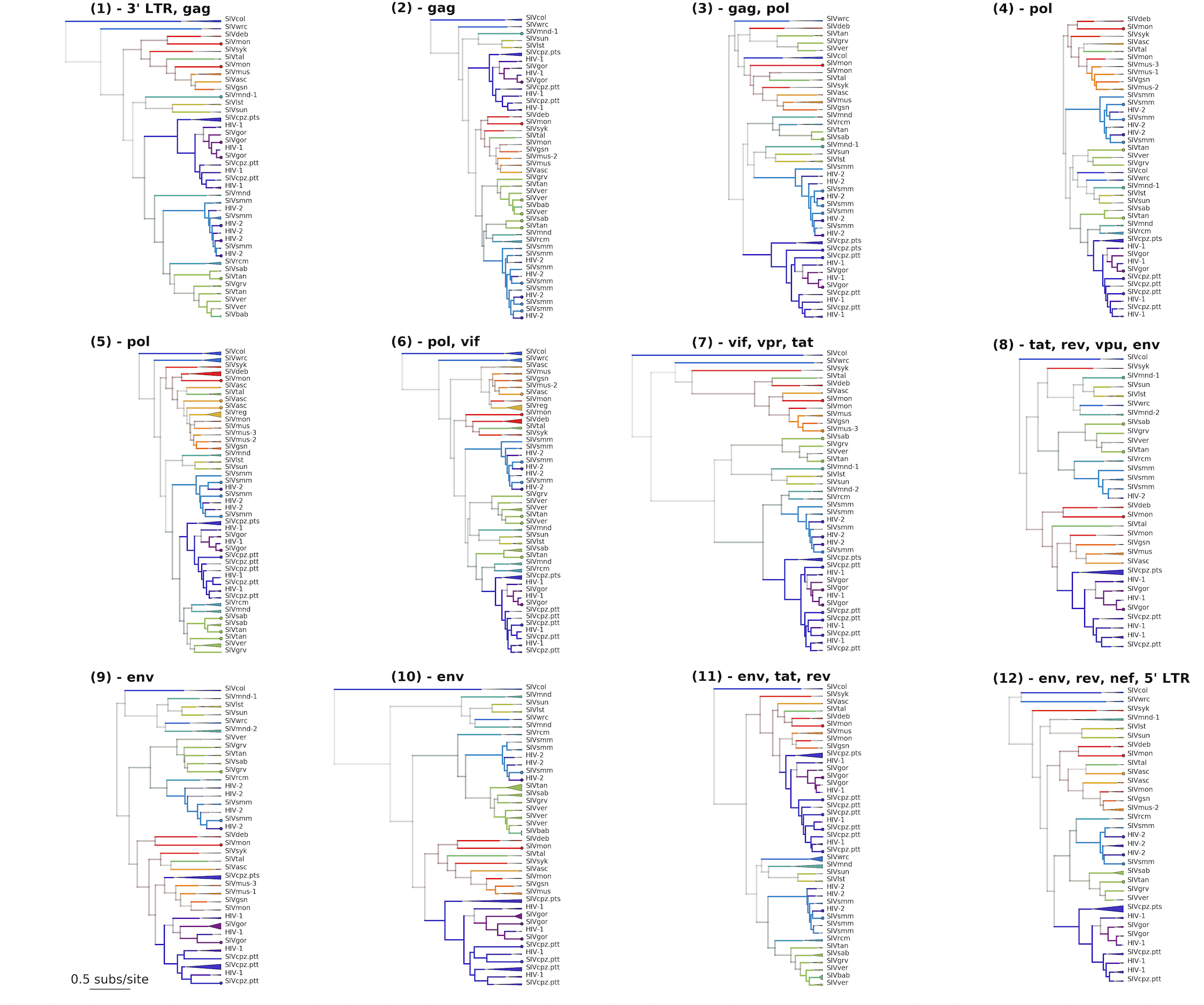

Supplement: S12 Fig — Tips are color coded by known host state; branches and internal nodes are color coded by inferred host state, with color saturation indicating the confidence of these assignments. Monophyletic clades of viruses from the same lineage are collapsed, with the triangle width proportional to the number of represented sequences. (TIF) [file ppat.1006466.s012.tif]
